# Self-Tuning Control Using an Online-Trained Neural Network to Position a Linear Actuator

**DOI:** 10.3390/mi13050696

**Published:** 2022-04-29

**Authors:** Rodrigo Hernandez-Alvarado, Omar Rodriguez-Abreo, Juan Manuel Garcia-Guendulain, Teresa Hernandez-Diaz

**Affiliations:** 1 Industrial Technologies Division, Universidad Politecnica de Queretaro, Carretera Estatal 420, El Marques 76240, Mexico; manuel.garcia@upq.edu.mx; 2 Center for Engineering and Industrial Development, Av. Playa Pie de la Cuesta No. 702, Queretaro 76125, Mexico; kathd.87@gmail.com

**Keywords:** neural network, self-tuning, PID, linear actuator, control

## Abstract

Linear actuators are widely used in all kinds of industrial applications due to being devices that convert the rotation motion of motors into linear or straight traction/thrust motion. These actuators are ideal for all types of applications where inclination, lifting, traction, or thrust is required under heavy loads, such as wheelchairs, medical beds, and lifting tables. Due to the remarkable ability to exert forces and good precision, they are used classic control systems and controls of high-order. Still, they present difficulties in changing their dynamics and are designed for a range of disturbances. Therefore, in this paper, we present the study of an electric linear actuator. We analyze the positioning in real-time and attack the sudden changes of loads and limitation range by the control. It uses a general-purpose control with self-tuning gains, which can deal with the essential uncertainties of the actuator and suppress disturbances, as they can change their weights to interact with changing systems. The neural network combined with PID control compensates the simplicity of this type of control with artificial intelligence, making it robust to drastic changes in its parameters. Unlike other similar works, this research proposes an online training network with an advantage over typical neural self-adjustment systems. All of this can also be dispensed with the engine model for its operation. The results obtained show a decrease of 42% in the root mean square error (RMSE) during trajectory tracking and saving in energy consumption by 25%. The results were obtained both in simulation and in real tests.

## 1. Introduction

Electric linear actuators are a class of synchronous servomotor, which are used where it is required to exert large forces and large displacements. According to their model, these actuators create movements in a straight line, which is slow. Currently, there are positioning studies, such as [1] which present results on a pneumatic linear peristaltic actuator in which they apply classical control laws such as PID, I-PD for control tuning using the Ziegler-Nichols rule. Their contribution is to mitigate the positioning error, and they compare it with conventional piston actuators. In [2], the authors present a model-based robust force control approach for pneumatic actuation systems. In their study, they heuristically apply the control law, in sliding mode control, to a double-acting cylinder. There are also studies of permanent magnet linear motors, such as the case of [3], where the authors present a small tubular motor that applies a control system by genetic algorithms. Actuators have a great application which is present in humanoid robots as in [4], where they have a direct-drive linear motor. They present the evaluation of a PID for positioning, acceleration, and force of the impact on the foot. In another work, they show the analysis of a hybrid linear actuator, such as [5], which is a pneumatic-electric actuator, solving the problem of position tracking and input allocation through convex optimization with a PD predictive control where the tuning was manual.

Although they are used in numerous applications, the most complex studies present classic control systems such as PID, PD plus gravity, first-order and higher-order sliding modes [6]. The investigation [7] shows a pneumatic actuator control focus on compensating the pressure of the actuator with sliding mode control. Its results are optimal since they focus on turning the actuator on and off, employing PWM, and using the RMSE to test the robustness of the control. The work [8] presents a hybrid control with a PID-enhanced slider mode controller for a Missile Electromechanical Actuator Systems, which contributes to the reduction of actuator vibrations. Similar work is presented in [9] where it introduces vibration reduction with predictive control. The research of [10] also presents a generalized control system where it attacks disturbances due to system mismatch.

The result of several studies on linear actuators has gradually improved the control results. However, the positioning error is still considerable in linear actuators. Positioning error occurs in other systems and has been addressed by different controllers. Some authors attack these problems using neural networks in non-linear plants, such as the works shown in [11,12]. The dynamic model of linear actuators is characterized by having a high non-linearity. Consequently, it is difficult to perform a positioning control in the face of unknown disturbances due to this characteristic.

Currently, studies of intelligent systems are used to control the position [13,14]. For example, the work [15] use a fuzzy system combined with classic control systems to fit the control signal according to the changes in dynamics. Another option within the intelligent control algorithms is Artificial Neural Networks (ANN), which can reduce positioning error [16,17]. Neural networks are helpful, as shown in [18,19] where they use the Recurrent Neural Networks with supervised learning to control non-linear systems with uncertainties. In addition, they present that the experimental part is carried out online, also as is the case of [20,21], evaluate networks with control to reduce uncertainty in plants. These works present a high computational level of network architecture when presented online. In order to reduce the amount of sample data and communication to have a better response online, widely used event-triggered control (ETC) methods have been extensively studied and gradually replaced the traditional scheme of periodic sample data such as [22].

In all positioning control applications, hysteresis and creep effects of electric actuators have been shown to significantly degrade system performance and even system stability, as seen in [23,24]. In general terms, an Artificial Neural Network performance depends strongly of the training stage and some studies present extremely long training periods. There are also combinations of PID controls and an intelligent system designed to self-tuning the gains of different systems, such as non-linear systems [25,26]. Other works present studies with genetic algorithms in combination with a PID such as [27], which attack the settling time of trajectory tracking and other authors present combinations with a high computational level when using genetic algorithms and fuzzy controls such as [28]; in the same way, combinations of Fuzzy Controls are presented, as in the case of [29], where disturbances are attacked with a combination of Active Perturbation Rejection Control with Takagi-Sugeno Proportional Derivative Fuzzy Control called ADRC-PDTSFC. There are other methods where controllers are adaptive which are playing a key role in intelligent control systems, using them as complex systems where plant parameters are unknown, and are subject to high uncertainties as is the MRAC method [30], this method has the ability to predict and can change according to the change of the plant, the main challenges of working with MRAC are that it requires an understanding of how the system actually operates, machine learning techniques have also shown great promise in the field of adaptive control. Methods such as neural networks, reinforcement learning, and fuzzy logic have been presented with plants where the response time must be very high, as in the case of [31], where the authors proposed a method of automatic tuning of gains of a PID control online, utilizing neural networks before highly non-linear systems, mainly attacking the unknown disturbances which occur in a hostile environment. It also presented the combination of a genetic algorithm with neural networks [32] which reduces the response error using a PID of auto-adjustment of gains. As can be seen, the use of neural networks is widely helpful to attack non-linear systems with uncertainties in the face of changing environments.

Multiple intelligent systems that solve the positioning problem are studied in the previous works. However, none of the papers exhibited an online training stage, which offers an advantage in implementation and execution time since it is well known that the training stage is the most computationally expensive. Therefore, we present the study of the position control of an electric linear actuator in real-time and attack the sudden changes of loads and limitation range by the control. It uses a general-purpose control with self-tuning of its gains. The control system tracked the trajectory even if the system model is unknown, which can deal with the essential uncertainties of the actuator and suppress disturbances, as they can change their weights to interact with changing systems.

The organization of the document presents Section 2 the Background, the model is expressed mathematical Dc motor with rotational load and Neural Network Self-Tuning PID Controller Design. In Section 3 Implementation and control execution; Section 4 presents results; and finally, Section 5 shows the final observations (conclusions).

## 2. Background

**Figure 1:**
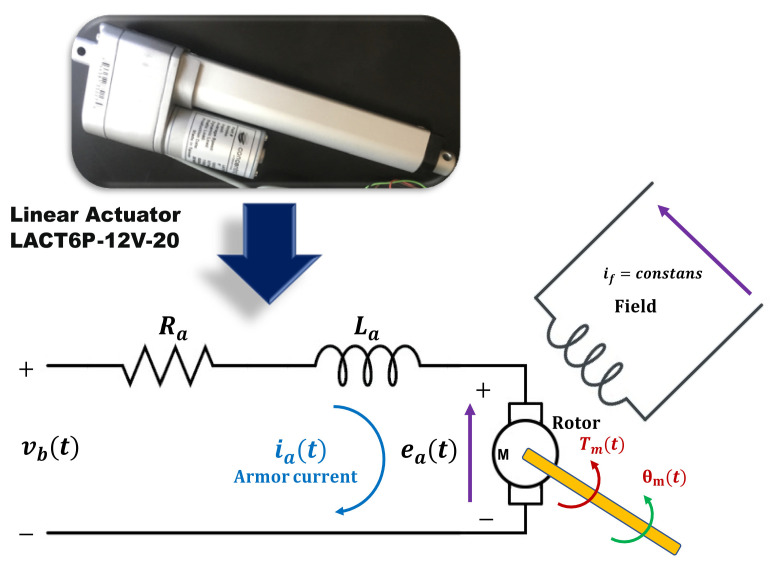
Scheme of linear electric actuator mechanism.

The structure of the direct current linear actuator is shown in Figure 1, which is used in a variety of heavy-duty applications such as electric wheelchairs model LACT6P-12V-20. The motor has a 20:1 reduction gearbox that can drive a dynamic load of 50 kg and a maximum speed of 1.3$\frac{\mathrm{cm}}{s}$.

### 2.1. DC Motor with Rotational Load

The diagram of the electric motor is presented in the following Figure 1. It can be seen that permanent magnets generate a magnetic field. In the armature, there is a current $i_{a}\left(t\right)$, which circulates through the magnetic field at right angles and detecting a force as expressed in Formula (1).

(1) $$F=BI*i_{a}\left(t\right)$$

where *B* is the magnetic field intensity, *I* is the inductance of the motor. From mesh analysis, Formula (2) is obtained.

(2) $$v_{b}\left(t\right)=R_{a}i\left(t\right)+L_{a}\frac{di_{a}\left(t\right)}{dt}+E_{a}\left(t\right)$$

By clearing from expression (2), Formula (3) is obtained.

(3) $$\frac{di_{a}\left(t\right)}{dt}=\frac{v_{b}\left(t\right)-R_{a}i\left(t\right)-Ea\left(t\right)}{L_{a}}$$

where $E_{a}$ (*t*) is a generated voltage that results when the armature conductors move through the field flux established by the field current $i_{f}$.

The equation of the mechanical part is generated from the torque generated by the motor, and which is represented in Formula (4).

(4) $$T_{m}\left(t\right)=J\frac{d\omega }{dt}+B\omega \left(t\right)$$

Clearing the derivative of the angular velocity of the previous equation gives Formula (5)

(5) $$\frac{d\omega (t)}{dt}=\frac{T_{m}\left(t\right)-B\omega \left(t\right)}{J}$$

where $T_{m}\left(t\right)$ is the motor torque. *B* is the coefficient of friction equivalent to the motor and the load mounted on the motor shaft. *J* is the total moment of inertia of the rotor, and the load in relation to the motor shaft. ω(t) is the angular velocity of the motor. The back electromotive moment is assumed to exist in a proportional relationship, $K_{a}$, between the voltage induced in the armature and the angular velocity of the motor shaft as shown in Formula (6).

(6) $$E_{a}\left(t\right)=K_{a}\omega \left(t\right)$$

In the same way, the electromechanical relationship that establishes that the mechanical torque is proportional to $K_{m}$, to the electric current is exhibited in Formula (7).

(7) $$T_{m}\left(t\right)=K_{m}i\left(t\right)$$

The above equations describe the behavior of the direct current motor over time. However, they can also be analyzed in the Laplace domain. For this, the Laplace transform is applied, and the Formulas (8)–(11) were obtained:

(8) $$Lsi\left(s\right)=v\left(s\right)-Ri\left(s\right)-E_{a}\left(s\right)$$

(9) $$\omega \left(s\right)=T_{m}\left(s\right)-B\omega \left(s\right)$$

(10) $$E_{a}\left(s\right)=K_{a}\omega \left(t\right)$$

(11) $$T_{m}\left(s\right)=K_{m}i\left(s\right)$$

Formula (12) represents the transfer function that relates the angular velocity and the voltage. On the other hand, the Formula (13) shows the relationship between the position of the rotor and the voltage applied to the motor, for more details it is described in [10].

(12) $$\frac{\omega (s)}{v(s)}=\frac{K_{m}}{LJs^{2}+\left(RJ+LB\right)s+\left(RB+K_{m}K_{a}\right)}$$

(13) $$\frac{\theta (s)}{v(s)}=\frac{K_{m}}{s(LJs^{2}+\left(RJ+LB\right)s+\left(RB+K_{m}K_{a}\right))}$$

**Figure 2:**
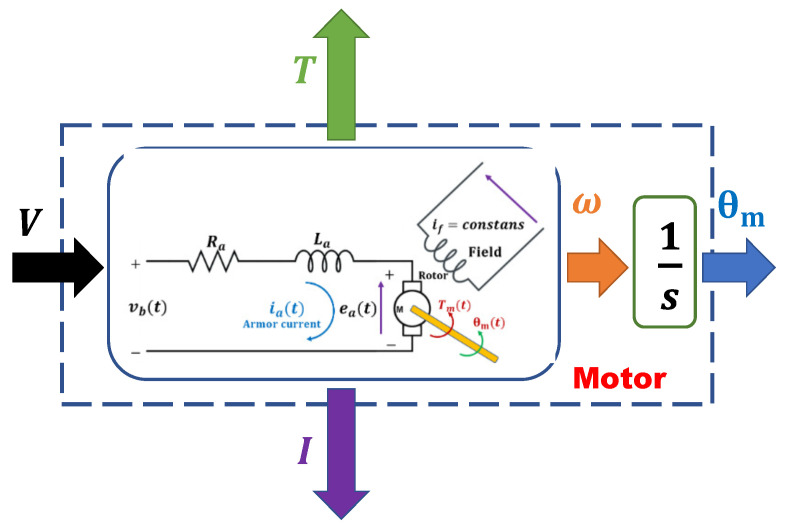
Scheme diagram of the DC motor.

The transfer functions (12) and (13) are represented in Figure 2.

The heuristically obtained values used for this model are: *J* = 0.01$\frac{{\mathrm{Kgm}}^{2}}{s^{2}}$, *B* = 0.1 Nm, $K_{m,a}=0.01$, R=1Ω, *L* = 0.5H. The Table 1 shows a summary of the variables used for the engine model and their description.

**Figure 3:**
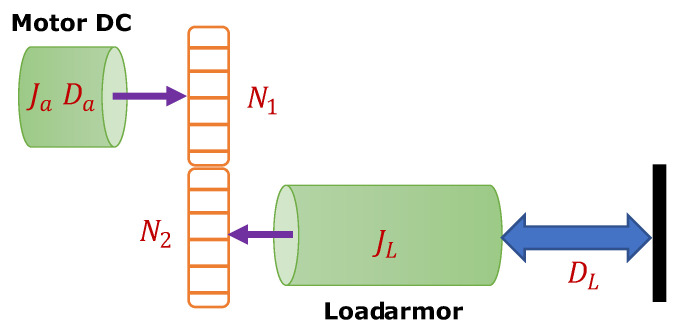
Relationship between rotational motor and linear displacement.

Finally, the angular relationship (motor advance angle) with the motor displacement (endless screw) must be obtained since connecting the motor with the load is required. As seen in Figure 3, the motor with inertia $J_{a}$, the motor damping $D_{a}$, $J_{L}$ represent the armature that moves the load formed by the inertia, where $D_{L}$ is the damping of the load.

**Figure 4:**
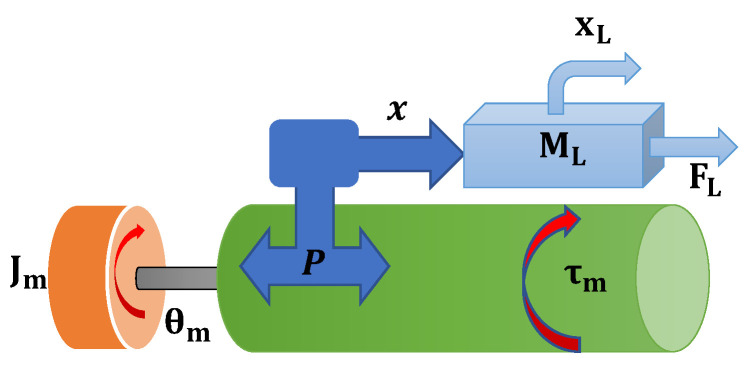
Transformation of rotational motion to linear motion.

The relation of $N_{1}$ and $N_{2}$ is considered to obtain the inertia relation $J_{m}=J_{a}+J_{L}{\left(\frac{N_{1}}{N_{2}}\right)}^{2}$ with the damping relation $D_{m}=D_{a}+D_{L}{\left(\frac{N_{1}}{N_{2}}\right)}^{2}$. Therefore, the relationship of the angular conversion with the linear displacement can be observed in Figure 4.

Where the motor angle is represented by Formula (14).

(14) $$\theta_{m}\left(t\right)=K_{m}i\left(t\right)$$

### 2.2. Neural Network Auto-Tuning PID Controller Design

**Figure 5:**
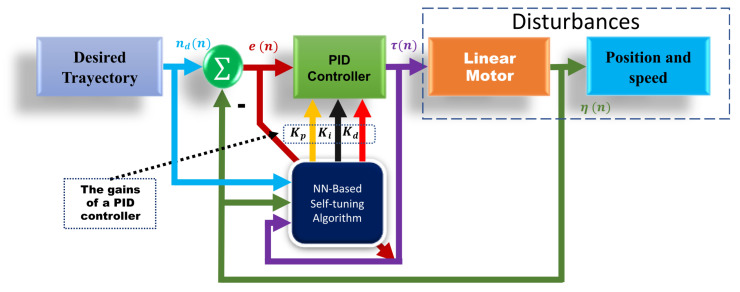
PID controller auto-tuning diagram.

An Auto-tuning PID controller architecture with neural networks is shown schematically in Figure 5. This architecture uses a feedback control unit and a Recurrent Network with Supervised Learning and Delayed Structure to adjust the parameters of the PID controller (how and why the combination was selected can be seen in [33]). When system parameters vary, the neural network can timely correct the PID controller parameters and properly choose parameters for the input layer based on the problem and system architecture.

In general, any system input signal $\left(\eta_{d}\left(n\right)\right)$, error signal (e(n)), you can choose the controller output (τ(u)) and the system output signal (η(n)) as the input layer parameters. However, the network outputs are the PID controller gains. The self-tuning control architecture with neural networks for a PID can be described in discrete time as follows:

(15) $$\begin{array}{c} \tau =\tau \left(t-1\right)+K_{p}\left(e\left(t\right)-e\left(t-2\right)\right)+K_{I}\left(e\left(t\right)\right)+K_{d}\left(e\left(t\right)-\left(e\left(t-2\right)\right)+e\left(t-3\right)\right) \end{array}$$

**Figure 6:**
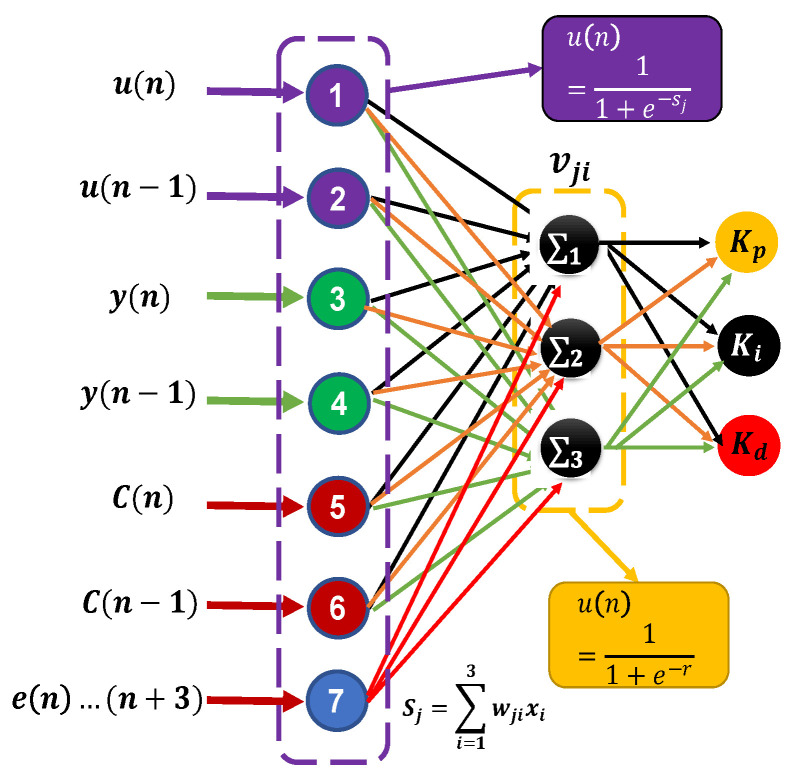
Block diagram of the implemented NN.

The method to be developed in the present work calculates the parameters of the controller online (proportional $K_{p}$, integral $K_{i}$ , derivative $K_{d}$) to give a better panorama is seen in Figure 6 where the entries that the neural network must have are appreciated. However, to determine these parameters, as is known when working with a neural network, it is necessary to have the input and output patterns already known a priory. In this case, the output patterns are not known in advance (the output patterns are not known $K_{p}$, $K_{i}$, $K_{d}$); hence, the combination of the selection of the two neuron methods for tracking trajectories, where the Recurrent Network with Supervised Learning is used in discrete time here for the PID control in discrete time Equation (15), presenting to the network a set of patterns, together with the desired output or target, and iteratively adjusting its weights until the output tends to be the desired output, and the network with delayed structure this type of structure tends to minimize the difference between the desired signal (input signal at the moment *n*) and the output of the neural network that will be a certain value obtained with values of the signal, by adjusting the current signal from the previous values.

The type of network used for automatic adjustment is the combination of a Recurrent Network with Supervised Learning and Delayed Structure see [31], where part of the mathematical development of the neural network is shown.

The basic structure of this type of network is shown in Figure 6. According to the previous figure, u(n) and u(n−1) corresponds to the reference input signal (desired trajectory), y(n) and y(n−1) is the reference output signal (real trajectory), C(n) and C(n−1) correspond to the control signal, e(n)…e(n+3) is the error signal up to 3 steps back for a better response to changing environments according to the use of neural networks, the weights of the hidden layer is $W_{ji}$ and the weights of the output layer is $v_{ji}$.

The structure of the proposed network was as follows: seven neurons in the input layer, three in the hidden layer, and three in the output layer. This design was the one that best adapted to the problem. The network was trained online and implemented the communication of the feedback sensor and the other electronic components (low computational level).

It was also possible to compare the work with [34] which presents a neural network approach for the control of in-line self-tuning and real-time implementation for a flexible micro-actuator. In addition, Reference [35] uses a self-tuning PID control and presents a high computational level in both cases, this to its neural network structure.

The error function for expressing better learning is:

(16) $$J=\frac{1}{2}e^{2}\left(k\right)=\frac{1}{2}{\left(u\left(n\right)-y\left(n\right)\right)}^{2}$$

where u(n) is the target input value (desired trajectory), and y(n) is the actual output value (real trajectory). As seen in Figure 5, we know that a self tuning PID controller will be designed to perform the force position tracking task. A gradient descent method has been used to minimize the error function see [36]. Where the learning rate η=0.01, and the inertia factor α=0.5.

Discrete time PID controller combined with neural network is obtained

(17) $$\frac{\partial u(k-1)}{\partial O_{k}}=\left\{\begin{array}{c} e(k-1)-e(k-2)\\ e(k-1)\\ e(k-1)-2e(k-2)+e(k-3) \end{array}\right.$$

where $O_{1}$ is gain $K_{p}$, $O_{2}$ is gain $K_{i}$ and $O_{3}$ is gain $K_{d}$.

Where $e_{y}\left(k\right)=y_{r}\left(n\right)-y\left(n\right)$. Additionally, it is assumed that time has been discretized by using small intervals of time equal to space [31], as exhibit the Formula (18).

(18) $$\frac{\partial E(t)}{V_{j}}=\frac{\partial E(t)}{\partial e_{y}}\frac{\partial e_{y}}{\partial e_{u}}\frac{\partial e_{u}}{\partial K_{i}\left(t\right)}\frac{\partial K_{i}\left(t\right)}{\partial r_{j}}\frac{\partial r_{j}}{\partial V_{j}}$$

The partial derivatives of the function E(t) with respect to the weighting coefficients $W_{ji}$, can be obtained by applying the chain rule again (Formula (19))

(19) $$\frac{\partial E(t)}{V_{j}}=\frac{\partial E(t)}{\partial e_{y}}\frac{\partial e_{y}}{\partial e_{u}}\frac{\partial e_{u}}{\partial K_{i}\left(t\right)}\frac{\partial K_{i}\left(t\right)}{\partial r_{j}}\frac{\partial r_{j}}{\partial V_{j}}\frac{\partial h_{j}}{\partial S_{j}}\frac{\partial s_{j}}{\partial W_{ji}}$$

From the previous expression, the weights are obtained as shown in Formulas (20) and (21).

(20) $$V_{j}\left(t+1\right)=V_{j}\left(t\right)+\left(\Delta \frac{\partial e_{y}}{\partial e_{u}}\right)\partial^{1}h_{j}$$

(21) $$W_{ij}\left(t+1\right)=W_{j}\left(t\right)+\left(\Delta \frac{\partial e_{y}}{\partial e_{u}}\right)\partial_{j}^{2}x_{j}$$

where δ is the learning coefficient, $V_{j}\left(t+1\right)$ is the vector of weights of the output layer, $W_{ji}\left(t+1\right)$ is the vector of weights of the hidden layer and $\left(\frac{\partial e_{y}}{\partial e_{u}}\right)\partial^{1}h_{j}$ is the equivalent gain.

## 3. Implementation and Control Execution

In this section, the results obtained are discussed. Two sets of experiments are presented. One consists of the conventional PID controller, and the other consists of the control proposed in this article. Both controls were evaluated under the same conditions to test their performance in change positioning. A comparative mathematical analysis of position tracking (simulation and implemented system) and power consumption is provided.

The proposed self tuning PID control was evaluated through a four retro 12 V 20:1 Linear Actuator. The motor has a 20:1 reducer that gives the actuator a dynamic load capacity of 110 pounds (50 kg) and a maximum speed of 0.5 in/s (1.3 cm/s). An embedded plate was used as a protection and data acquisition system to manipulate the positioning of the linear actuator (feedback system) using the obtained data values from the internal actuator potentiometer, which received readings of 1024 bits. The LabVIEW software was used to monitor and store the data for the algorithm proposed in this article was programmed as the conventional control.

### 3.1. Experimentation in Simulation

**Figure 7:**
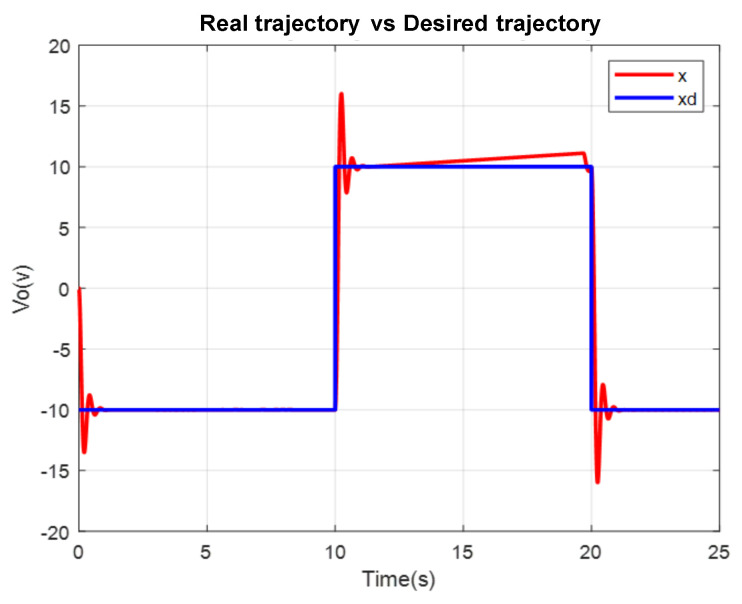
Conventional PID Real versus desired trajectory (position change).

**Figure 8:**
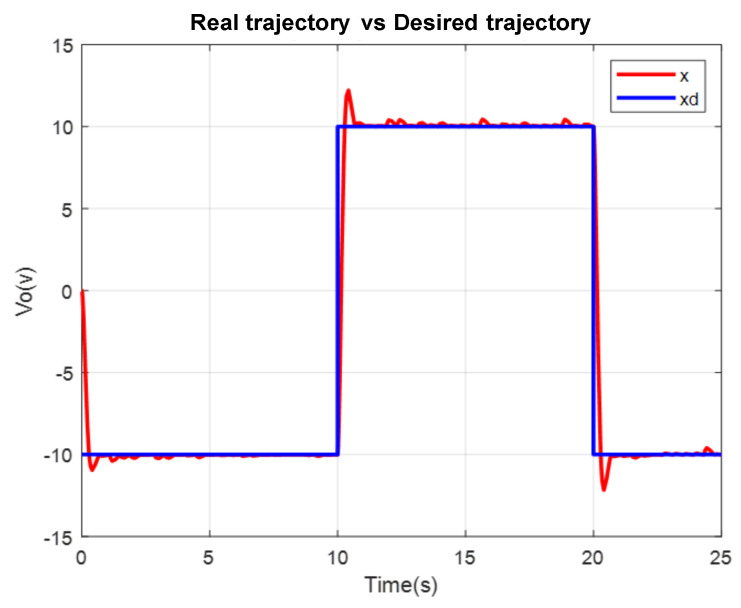
Self tuning control: real versus desired trajectory (position change).

A comparison in the simulation of the conventional PID control and the self-tuning PID control must be made to observe the performance of the system. However, the conventional control in Figure 7 shows a change when the disturbance increases in Figure 8. This is observed in the following figures. The simulation was developed with a variable step ODE45 and a step from −10 to 10 with a disturbance in time 10 s. Matlab/Simulink software was used, which had a simulation time of 25 s, taking the gains for conventional control of $K_{p}=161$, $K_{i}=21$ and $K_{d}=275$ (gains obtained heuristically)

**Figure 9:**
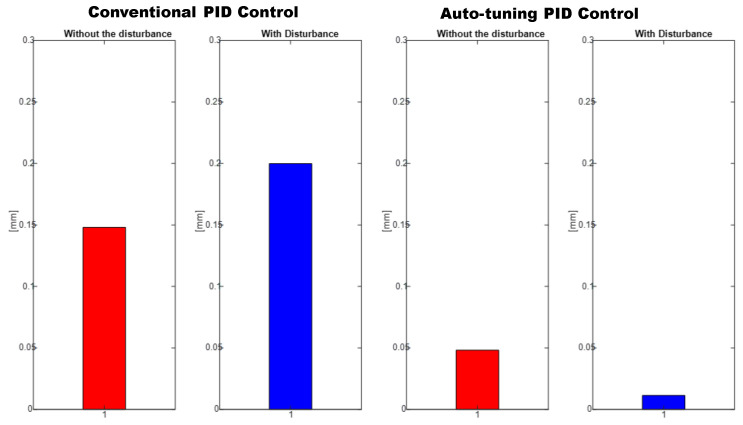
RMSE: Conventional PID controller (**left**) vs. auto-tuning PID (**right**).

To verify the performance of the conventional PID control and the proposed control system, since not much difference is appreciated in the Figure 9, the mean square error of the trajectory is applied. It can be observed how the proposed system has a better response.

**Figure 10:**
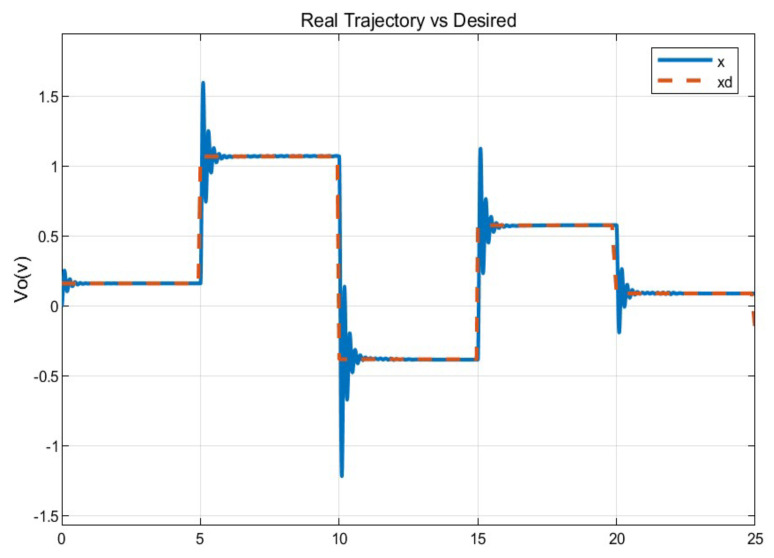
Auto tuning PID gain simulation and implemented in conventional PID control.

To prove that the gains obtained from the self-tuning PID control are better than the heuristically tuned control, Figure 10 shows the gains obtained with the proposed PID and is executed, where the correct operation is appreciated, and it can be observed that the proposed control gets good results; it should be noted that such gains are only of a controlled disturbance since the conventional control only works in a working range (design range of the linear actuator PID).

The initial values that the neural network takes are the following: neuron weight coefficients *W* initial values of −1, 0 and 1, output neuron weight coefficients *V* initial values −1, 0 and 1, and initial values of the variables $K_{p}$, $K_{i}$ and $K_{d}$ of 0.

### 3.2. Experimentation

**Figure 11:**
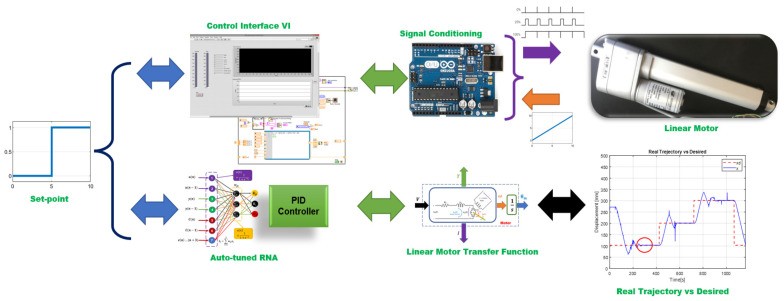
Block diagram for parameter estimation.

For the physical implementation in a linear motor, the block diagram shown in Figure 11 was applied.

**Figure 12:**
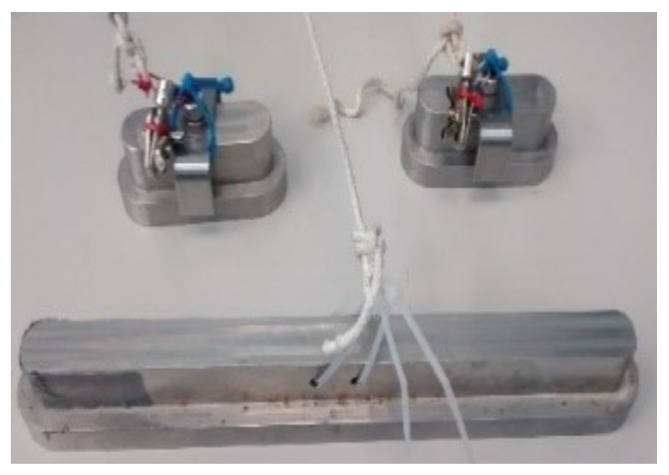
Loads (Perturbation).

The tasks assigned for evaluating the positioning of the linear actuator, two configurations are presented. The first evaluation was with the conventional PID control tuning. The second was considered the self-tuning PID control proposed in this article. A comparative analysis is provided between both control systems compared to the root mean square error of the trajectory. Both methods were subjected to the same disturbance conditions: weight increases of 9 kg in total as shown in Figure 12.

## 4. Results

The controls were evaluated under the same conditions; first, a data capture was made at different positions of the actuator; then, the disturbances increased the weight increase (disturbance).

Gains from conventional PID control were obtained by NN. The actuator was set to a reference point using the self tuning PID controller. Once the actuator reached the stability and gains of the PID, calculated by the NN, became stationary, these gains were used in the conventional PID such as $K_{p}$, $K_{d}$, and $K_{i}$. This is the procedure used for tuning the conventional PID gains. It is important to note that gains remained constant throughout the experiment once the conventional PID was adjusted, even when disturbances occurred, unlike the self tuning PID controller, where gains were dynamically changed to achieve better performance.

**Figure 13:**
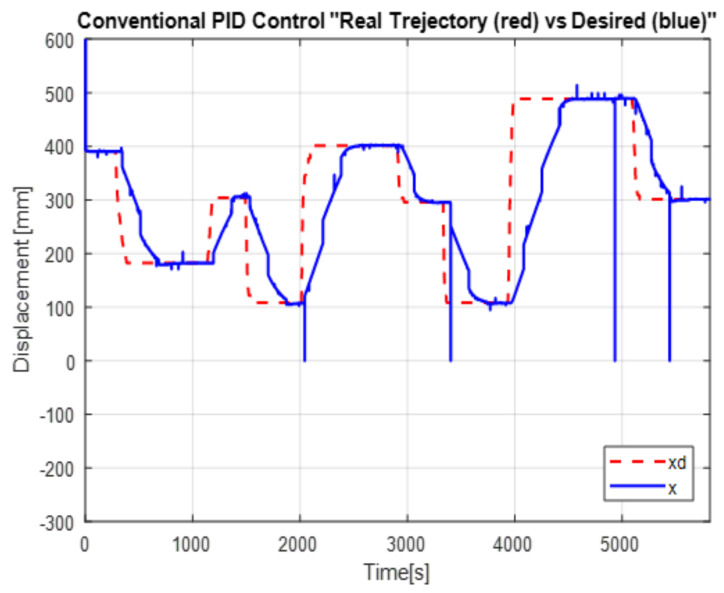
Real vs. desired trajectory (position change) of the conventional control.

**Figure 14:**
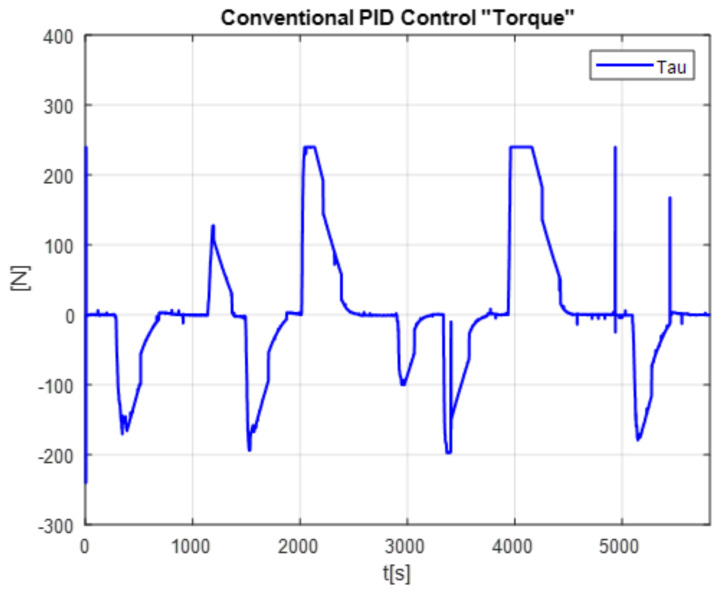
PID Controller without the disturbance.

The Figure 13 shows the desired path (solid line) against the actual path (dotted line), and the Figure 14 shows the control signals

**Figure 15:**
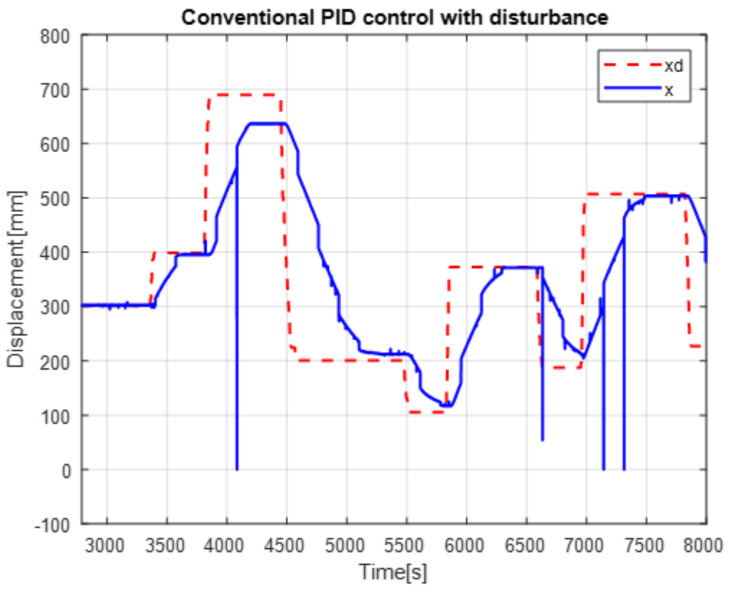
Real vs. desired trajectory (position change) of the auto tuning control.

**Figure 16:**
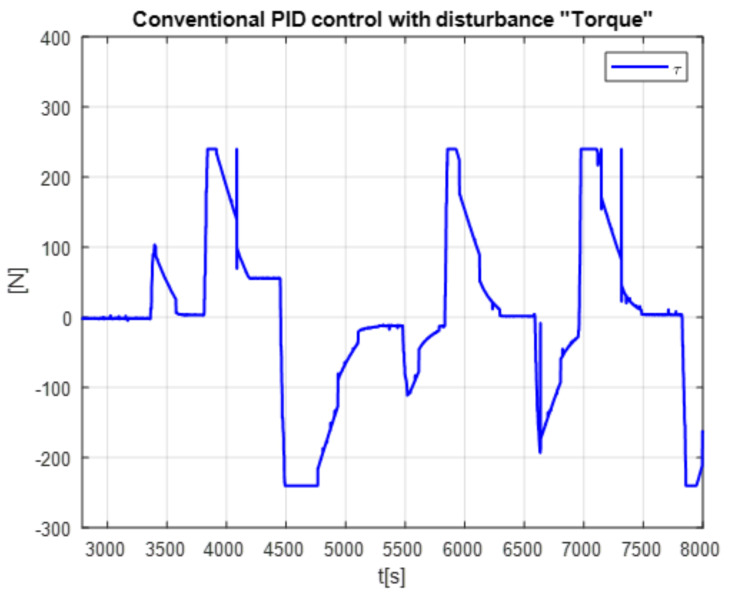
Disturbance free self tuning PID control.

The second experiment increases the perturbation, can be seen in Figure 15, as the control no longer reaches the desired trajectory xd, In the same way, the behavior of position change and the control reaction is presented Figure 16.

The real and desired trajectories are presented for the conventional PID control without disturbance Figure 13 and with disturbance Figure 15. It is observed for the first case (without disturbance), the output signal “real trajectory *x*” converges to the desired xd signal. On the other hand, the control no longer converges when the disturbance (weight gain) increases. This problem must be attacked with new gains for the PID control. The proposed system estimates the gains without a training stage. This provides an adaptable system non based on previous data.

**Figure 17:**
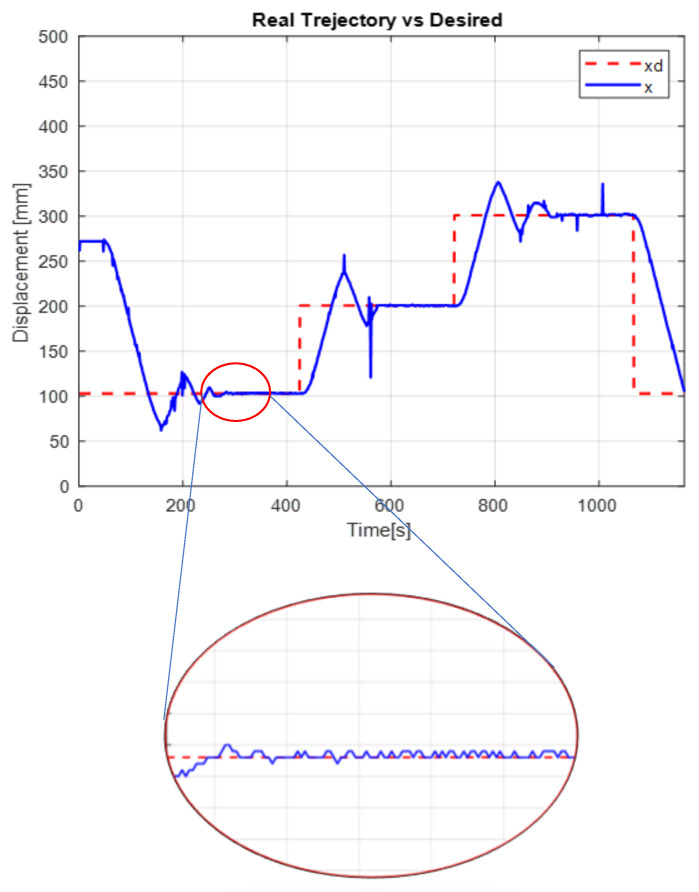
Real vs. desired trajectory with disturbance.

Figure 17 displayed the results for the autotuning PID control showing the actual versus the desired trajectory. The evaluated task was that perturbations were increased at different times; this can be seen in the steps as the “x output” converges to the “xd input”. It is worth mentioning that the disturbances were implemented randomly. The control reaches perceivable levels but still converge again.

**Figure 18:**
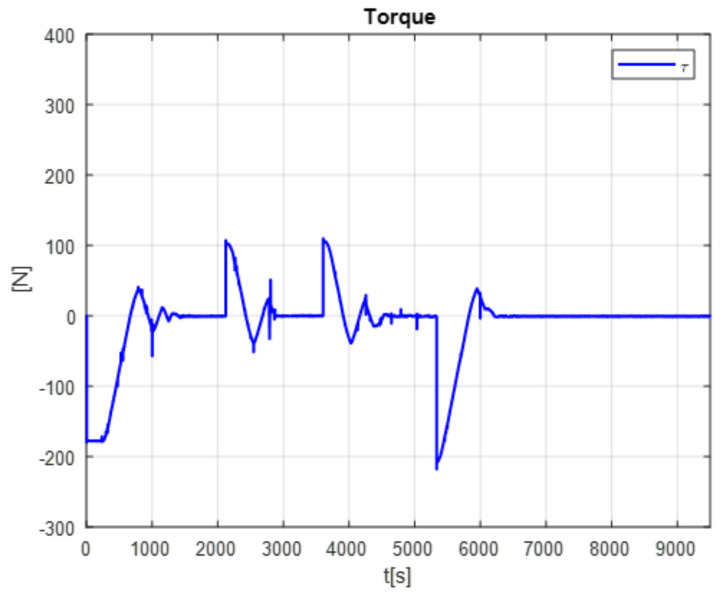
Self tuning control with disturbances.

The control reaction is shown in the Figure 18.

The following were captured concerning the currents: the static current 0.08 A with weight; current down 1.06 A, current up 0.85 A. It could be observed that the current varies up and down, but when it finds the point (point desired), the current is the same as when it has no weight, which is 0.08 A.

As can be seen from the results obtained in evaluating the different applied tasks, a conclusion cannot be drawn. Knowing which is the best control for the increase in disturbances for them is evaluated employing the mean square error (RMSE) (Formula (22)).

(22) $$RMSE=\sqrt{\frac{1}{N}\sum_{i=1}^{N}{\left(y_{i}-\hat{y}\right)}^{2}}$$

where $y_{i}$ is the actual actual output value and $\hat{y}$ predicted output value and *N* is the number of observations.

**Figure 19:**
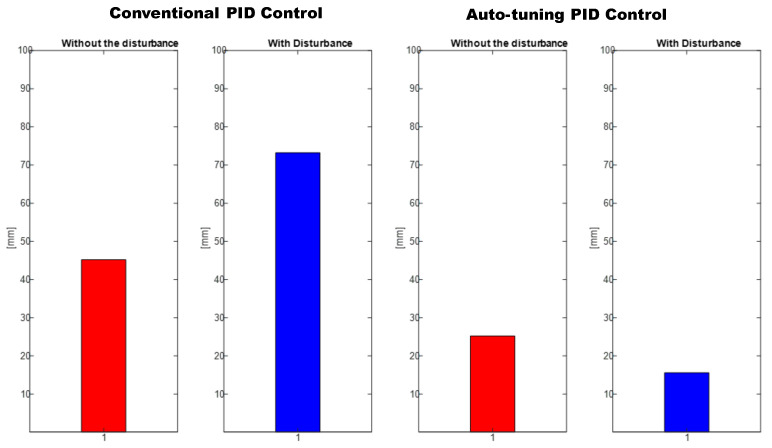
RMSE: Conventional PID Controller (**left**) vs. auto-tuning PID (**right**).

As mentioned above, the RMSE was used to evaluate trajectory tracking (Figure 19), where it can be seen that the control proposed in this document has a better convergence in the increase of weights (disturbances); therefore, we can conclude that the self-tuning PID control has a better response to the change of its dynamic parameters.

The positioning is displayed in Figure 13, Figure 15 and Figure 17. The conventional PID control response bars are observed on the left side. It is visualized that without load, the control has a better response than when the load is increased (red bar without load and blue with disturbance); the error increases by 42%, and energy consumption rises 25%. On the other hand, the bars on the right are the control responses proposed in this article. It is observed that even without load, they are already below the conventional control system, and when the load is increased, the positioning error and control response decrease even more. Therefore, energy consumption changes when it drops by 38%, where there is a difference in energy consumption of 13%. This phenomenon is observed in Figure 14, Figure 16 and Figure 18 where the convergence of the control systems is appreciated.

Finally, the comparison of the self-tuning gain method of the PID controller implemented for a DC motor with applications for hospital beds and chairs is made. The Table 2 shows the comparison of titles similar to this work, which refer to self-tuning of the gains of the PID controller by intelligent methods, such works attack the uncertainties, monitor the trajectory error making the control converge to the desired trajectory, and present simulations and prototypes applied online.

As seen in papers [17,31], although they are similar, they do not compare the control robust with themselves, just as a conclusion is not presented as to what happens when the network is not subject to disturbance, and one more conclusion is that, as it is well known that there are different methods for PID controller auto tuning; here with this work, it can be said that it does not matter how it is tuned, since they can find the ideal gains, but if their environment is changing then their gains are no longer the same. correct and the actual and desired trajectory may no longer converge.

**Figure 20:**
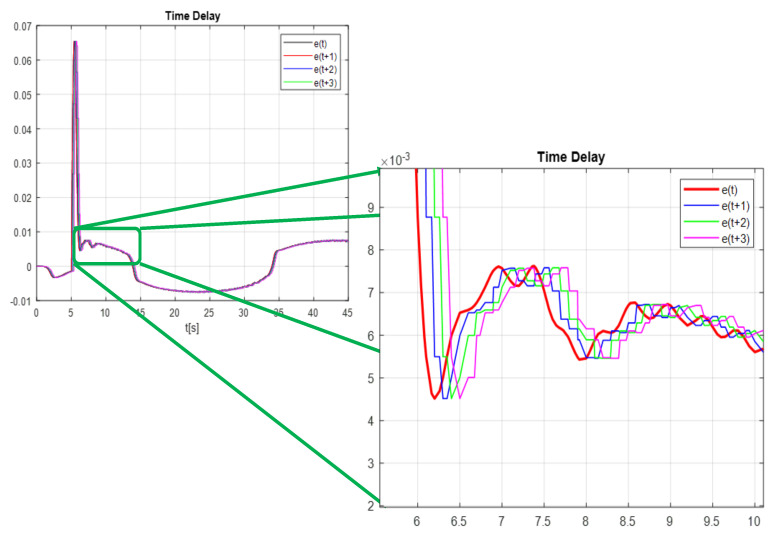
Time delay e(t),e(t+1),e(t+2),e(t+3).

The Figure 20 shows how the error advances up to three steps, so that the real trajectory converges to the desired trajectory before any disturbance impacts it, tests are made with more steps forward, but only up to the error (t+3) it is where the controller converges better, it is worth mentioning that further steps forward the controller enters gain saturation.

The present work found that the use of neural networks in combination with PID control, the more the control is demanded, the better the response, this is seen in Figure 13, Figure 15 and Figure 17 of trajectory tracking where it can be seen how an impulse destabilizes, but this is only for a moment since it instantly returns to the desired trajectory, also one of the greatest advantages of combining the PID controller with neural networks, since it is not necessary to know the model of the plant as it is, is it presents energy savings since when the control finds the desired path, the control enters into stable mode and considerably lowers the use of current and only activates when a disturbance is required or change; this occurs thanks to the fact that the network learns from its changing environments, since it recognizes disturbances and recognizes them if they come back to it; this is thanks to error handling, error signal up to 3 steps back (e+1,e+2,e+3) for better response to changing environments based on the use of neural networks. The experimental results were carried out for the validation of the controller and to see the robustness of the combination of a neural network with the PID control before its tuning of gains’ this was applied in this work which was to see how the conventional PID control acted with gains taken from the self-tuning PID control; although the conventional PID control has to be tuned online, so that it can deal with the disturbances that are increased or unknown disturbance, attacking this problem is why the neural network is increased for gain tuning to harden, so it would learn from its changing environments and be able to attack power consumption.

**Figure 21:**
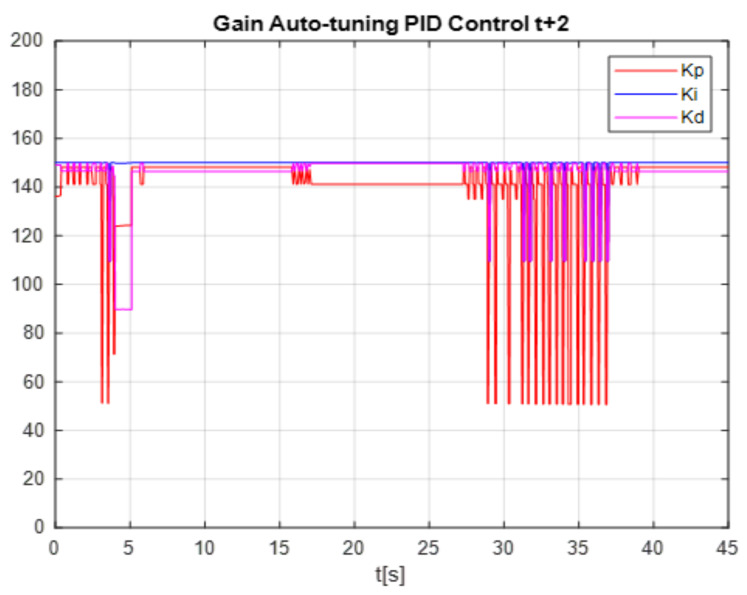
Gain Conventional PID Control.

**Figure 22:**
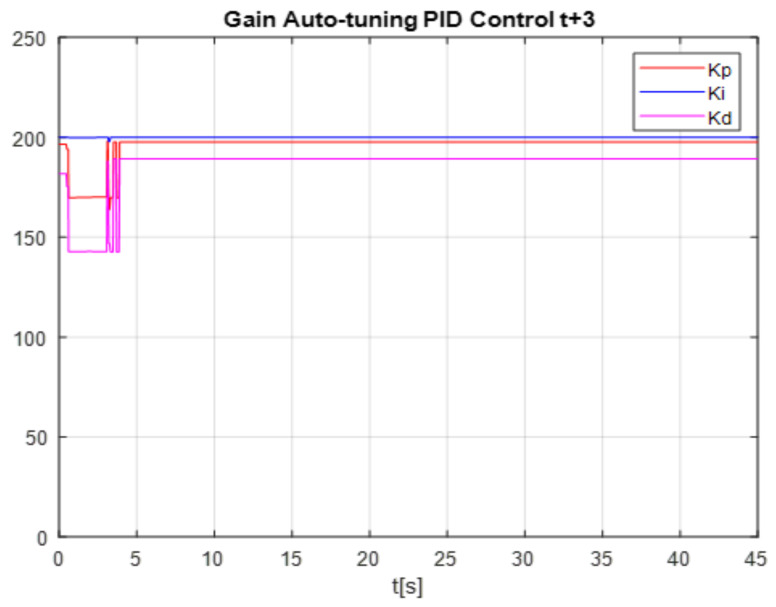
Gain PID auto tuning.

To see the differences between times t+2 and t+3, the comparison of the proposed control gains is shown, the gains obtained with time t+2 are shown in the Figure 21, it can be seen that at the beginning the network is learning (training) the time 2 to 5 s, then it becomes stable, but reaching the time of 27 s, the gains are destabilized again due to the change in disturbance, this occurs when applying the time of t+2. Unlike the time t+3, it can be seen in the Figure 22 that in the first 4 s the network is in training and its values after 4 s become linear and this is where it is clearly seen how applying times of t+3 is much better since that even if the disturbance is changed, the network learns from its environment.

## 5. Conclusions

This document presents the development of an algorithm for the automatic adjustment of PID control gains using neural networks. The kinematics and dynamics of the linear actuator model are developed. The conversion from rotation to linear is shown because it is an important part of the use of positioning control. The algorithm was evaluated to test its robustness to unknown disturbances (increase in weights), in which a linear actuator was used.

The performance of the algorithm was evaluated with the implementation of two tasks assigned to the tracking of trajectories, and to obtain a better conclusion, this document proposes a comparative analysis between the conventional PID control and the self-adjusting PID. One of the main advantages of this work is that the data obtained were obtained online, distinguishing it from previous works where training was conducted offline and with a large amount of data.

It is also presented that the computational cost is low due to the excellent combination of PID control and self-tuning of the controller gains since they quickly learn from the environment surrounding them. One of the advantages that it presents in implementing this algorithm is the low energy consumption. Once the real path connects with the desired path, the control system lowers its consumption and maintains the path until it changes. Its actuator dynamics, one of the advantages that could be observed with regard to energy consumption, are that when the disturbance is maintained, the network identifies it by instantly changing the gains according to the disturbance as shown in the results section.

At the end of the work, disadvantages were found which must be of extensive knowledge of programming algorithms, and in relation to the proposed method it was found that the neural network presents instability when it is in stationary state (network without disturbance requirements).

## Figures and Tables

**Table 1:** Variables used in the DC motor model.

Variable	Concept	Unit
$E_{a}$	Induced electromotive force	V
*B*	Coefficient of friction	Nm
*J*	Inertia	Kgm
$I_{a}$	Armature current	A
$K_{a}$	Electrical constant	−
$K_{m}$	Mechanical constant	−
$R_{a}$	Armor resistance	Ω
*v*	Armature voltage	V
ω	Angular velocity	$\frac{\mathrm{rad}}{s}$
$\theta_{m}$	Angular position	rad

**Table 2:** Work comparative. The symbol X represents a characteristic considered in the investigation. On the other hand, - represents an unconsidered characteristic.

Reference	Online	Error	Perturbations	PID Auto Tuning
Proposed method	X	e(t),e(t+1),e(t+2),e(t+3)	X	X
[11]	-	e(t+1)	X	-
[16]	X	e(t+1), e(t+2)	-	X
[17]	X	e(t+1)	X	X
[18]	-	e(t+1)	-	X
[19]	-	e(t+1)	-	X
[20]	X	e(t+1)	-	X
[21]	X	e(t), e(t+1),e(t+2)	-	X
[25]	X	e(t),e(t+1)	-	X
[26]	-	e(t+1)	-	X
[28]	-	e(t),e(t+1),e(t+2)	-	X
[31]	X	e(t),e(t+1),e(t+2)	X	X

## Data Availability

The data presented in this study are available on request from the corresponding author.
